# Supplementary material for: Classifying Obsessive-Compulsive Disorder from Resting-State EEG Using Convolutional Neural Networks: A Pilot Study
Source: Comput Psychiatr. 2026 Jan 16;10(1):1–17. doi: 10.5334/cpsy.149 (PMC12829452; doi:10.5334/cpsy.149)
Supplement: Supplementary file. — Supplement 1 and 2. [file cpsy-10-1-149-s1.zip › cpsy-149_zaboski/cpsy-149_zaboski-s1.docx]

**Supplement 1**

This supplement provides the detailed results of the epoch-level classification for the CNN, which formed the basis for the primary subject-level analysis presented in the main manuscript. When predictions were aggregated across all 2,277 test epochs from the 20 held-out subjects, the CNN’s overall classification accuracy reached 81.95%, and the ROC AUC was 0.86. Detailed performance metrics, including precision, recall, and F1-score, are presented in Table S1.

**Table S1**: *Overall Classification Performance of the CNN at the Epoch Level*

| **Class** | **Precision** | **Recall** | **F1-Score** | **Support** |
| --- | --- | --- | --- | --- |
| HC | 0.84 | 0.79 | 0.81 | 1148 |
| OCD | 0.80 | 0.85 | 0.82 | 1129 |
| **Accuracy** | - | - | 0.82 | 2277 |
| **Macro Avg** | 0.82 | 0.82 | 0.82 | 2277 |
| **Wtd Avg** | 0.82 | 0.82 | 0.82 | 2277 |

*Notes*: F1-Score: A single metric that balances precision and recall; support: Number of epochs belonging to that true class in the aggregated test sets; accuracy: Proportion of correctly classified epochs; macro avg: The unweighted average of precision, recall, F1 across both classes; weighted avg: The average of the metric across both classes, weighted by the support (number of true instances) for each class.

[Figure S1]

**Figure S1**: CNN Confusion Matrix, Receiver Operator Curve (ROC), and Precision-Recall Curve

The model's overall epoch-level performance characteristics are illustrated in Figure S1. The confusion matrix details the specific classification outcomes across all test epochs: 873 True Negatives (HC epochs correctly identified), 934 True Positives (OCD epochs correctly identified), 195 False Negatives, and 275 False Positives. The ROC and Precision-Recall curves further illustrate the model's performance on this epoch-level task.

[Figure S2]

**Figure S2**: Average Training and Validation Loss

Figure S2 shows the mean loss (solid lines) and standard deviation (shaded areas) across all 20 folds of the leave-one-subject-out cross-validation. The training loss (blue) rapidly converges, while the validation loss (red) also decreases, indicating model generalization. The high variance in the validation loss reflects the significant inter-subject heterogeneity in the data and the need for the subject-level analysis.

**Supplement 2**

This supplement provides the results of the epoch-level classification for the SVM, which formed the basis of comparison with the CNN. For comparison, we constructed an SVM trained on spectral power features (delta: 1 – 4 Hz, theta: 4 – 8 Hz, alpha: 8 – 13 Hz, beta: 13 – 30 Hz, gamma: 30 – 45 Hz). When aggregated across all 2,277 test epochs, the SVM's epoch-level accuracy was 49.01%, and the ROC AUC was 0.449. The full epoch-level model metrics are presented in Table 3, and the corresponding confusion matrix is shown in Figure S3.

**Table S2**. *Overall Classification Performance of the SVM at the Epoch Level*

| **Class** | **Precision** | **Recall** | **F1-Score** | **Support** |
| --- | --- | --- | --- | --- |
| HC (0) | 0.49 | 0.52 | 0.51 | 1148 |
| OCD (1) | 0.48 | 0.46 | 0.47 | 1129 |
| **Accuracy** | - | - | 0.49 | 2277 |
| **Macro Avg** | 0.49 | 0.49 | 0.49 | 2277 |
| **Wtd Avg** | 0.49 | 0.49 | 0.49 | 2277 |

*Notes*: F1-Score: A single metric that balances precision and recall; support: Number of epochs belonging to that true class in the aggregated test sets; accuracy: Proportion of correctly classified epochs; macro avg: The unweighted average of precision, recall, F1 across both classes; weighted avg: The average of the metric across both classes, weighted by the support (number of true instances) for each class.

[Figure S3]

**Figure S3**: SVM Epoch-Level Confusion Matrix, ROC, and Precision-Recall Curve
