# Supplementary figures and images for: Classifying Obsessive-Compulsive Disorder from Resting-State EEG Using Convolutional Neural Networks: A Pilot Study
Source: Comput Psychiatr. 2026 Jan 16;10(1):1–17. doi: 10.5334/cpsy.149 (PMC12829452; doi:10.5334/cpsy.149)

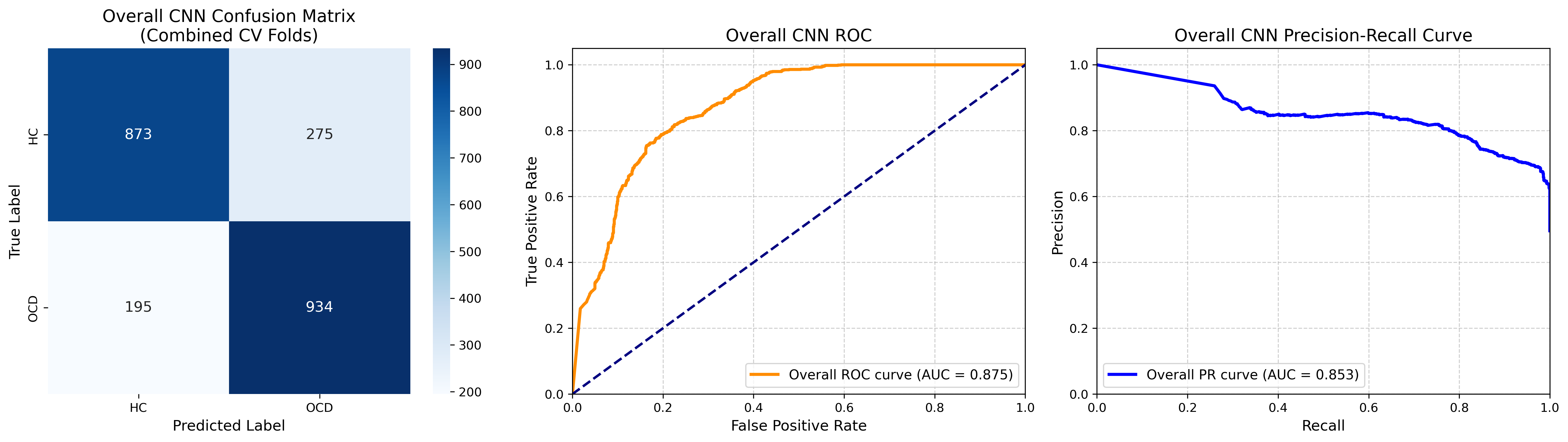

Supplement: Supplementary file. — Supplement 1 and 2. [file cpsy-10-1-149-s1.zip › cpsy-149_zaboski/Figure s1.png]

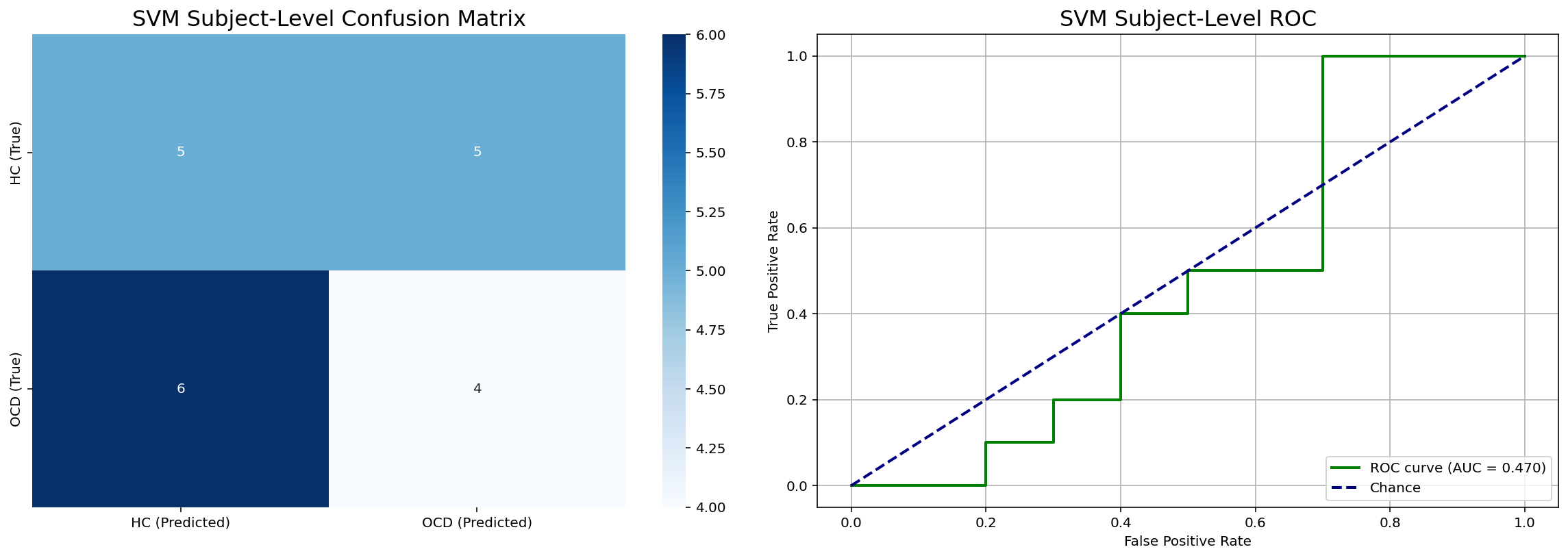

Supplement: Supplementary file. — Supplement 1 and 2. [file cpsy-10-1-149-s1.zip › cpsy-149_zaboski/Figure s2.png]

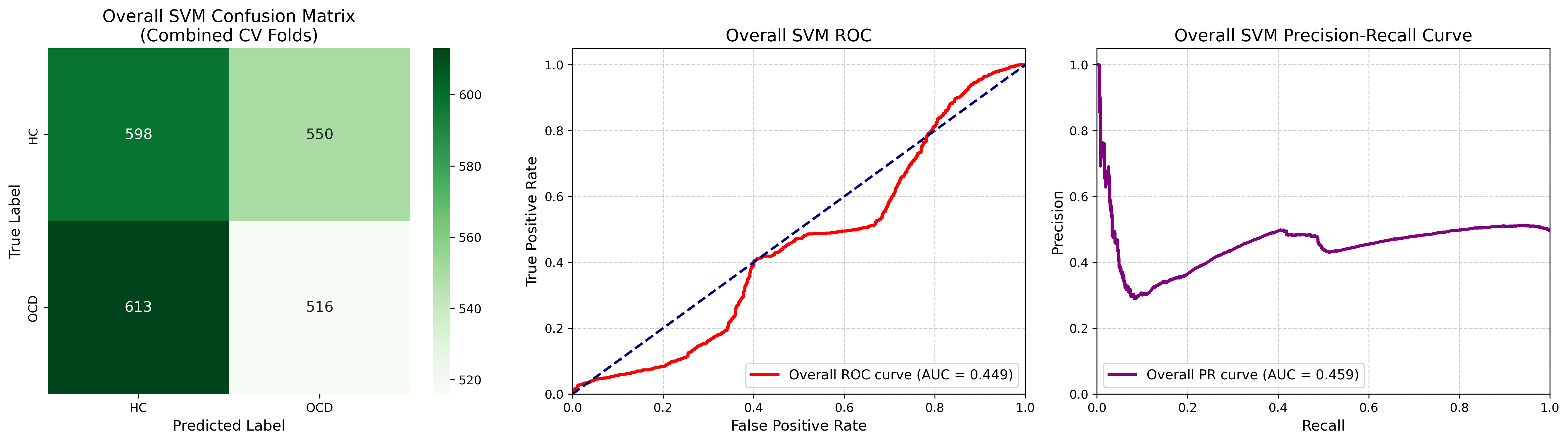

Supplement: Supplementary file. — Supplement 1 and 2. [file cpsy-10-1-149-s1.zip › cpsy-149_zaboski/Figure s3.png]
